# Quantitative insights into effects of intrapartum antibiotics and birth mode on infant gut microbiota in relation to well-being during the first year of life

**DOI:** 10.1080/19490976.2022.2095775

**Published:** 2022-09-29

**Authors:** Roosa Jokela, Katri Korpela, Ching Jian, Evgenia Dikareva, Anne Nikkonen, Terhi Saisto, Kirsi Skogberg, Willem M. de Vos, Kaija-Leena Kolho, Anne Salonen

**Affiliations:** a Human Microbiome Research Program, Faculty of Medicine, University of Helsinki, Helsinki, Finland; b Children’s Hospital, Pediatric Research Center, University of Helsinki, Helsinki, Finland; c Department of Obstetrics and Gynecology, University of Helsinki, and Helsinki University Hospital, Helsinki, Finland; d Clinic of Infectious Diseases, Jorvi and Helsinki University Hospital, Helsinki, Finland; e Laboratory of Microbiology, Wageningen University, Wageningen, the Netherlands; f Tampere University, Tampere, Finland

**Keywords:** Early-life microbiota, antibiotics, cesarean section, birth route, 16S rRNA gene amplicon sequencing, quantitative microbiota profiling

## Abstract

Birth mode and maternal intrapartum (IP) antibiotics affect infants’ gut microbiota development, but their relative contribution to absolute bacterial abundances and infant health has not been studied. We compared the effects of Cesarean section (CS) delivery and IP antibiotics on infant gut microbiota development and well-being over the first year. We focused on 92 healthy infants born between gestational weeks 37–42 vaginally without antibiotics (N = 26), with IP penicillin (N = 13) or cephalosporin (N = 7) or by CS with IP cephalosporin (N = 33) or other antibiotics (N = 13). Composition and temporal development analysis of the gut microbiota concentrated on 5 time points during the first year of life using 16S rRNA gene amplicon sequencing, integrated with qPCR to obtain absolute abundance estimates. A mediation analysis was carried out to identify taxa linked to gastrointestinal function and discomfort (crying, defecation frequency, and signs of gastrointestinal symptoms), and birth interventions. Based on absolute abundance estimates, the depletion of *Bacteroides* spp. was found specifically in CS birth, while decreased bifidobacteria and increased Bacilli were common in CS birth and exposure to IP antibiotics in vaginal delivery. The abundances of numerous taxa differed between the birth modes among cephalosporin-exposed infants. Penicillin had a milder impact on the infant gut microbiota than cephalosporin. CS birth and maternal IP antibiotics had both specific and overlapping effects on infants’ gut microbiota development. The resulting deviations in the gut microbiota are associated with increased defecation rate, flatulence, perceived stomach pain, and intensity of crying in infancy.

## Introduction

Human intestinal microbiota develops via successional stages during early life.^1^ The infant’s gut microbiota contributes to early immunological^2^ and metabolic programming that affect host health later in life.^3^ Transmission of maternal microbes to the offspring during birth plays a major role in the initial colonization.^1,4,5^ The mode of birth is a major determinant of neonates’ initial microbial exposure; the assembly and dynamics of intestinal microbiota in infants born via cesarean section (CS) versus vaginal delivery (VD) differ substantially,^1,6^ which has been suggested to mediate the negative long-term health outcomes related to CS birth.^7,8^

CS birth is not the only intervention influencing the neonate’s early colonization. Intrapartum (IP) antibiotic prophylaxis is routinely administered to mothers undergoing CS to reduce the risk of post-cesarean maternal infection. In VDs, approximately 25% of mothers receive antibiotics to prevent neonatal group B Streptococcus (GBS) infection.^9^ Thus, antibiotics are used in a substantial number of deliveries in developed countries. Emerging evidence suggests that IP antibiotics affect the infant’s microbiota development over a period of several months.^10–12^

Previous studies utilizing next-generation sequencing (NGS) have characterized the infant gut microbiota using relative bacterial abundance. However, quantitative analyses indicate that early microbiota development entails major quantitative changes in total bacterial load as well as in individual taxa,^13,14^ and the effects of birth interventions on absolute bacterial profiles are unknown.^15^ In addition, existing studies addressing the effects of IP antibiotics on child health have largely focused on neonatal GBS infection.^16,17^ Recently, also their relationship to the atopic triad^18^ and body mass index^19^ up to 5 y age has been investigated.

To understand the impact of birth interventions on the gut microbiota in relation to health in early infancy, we followed the development of gut microbiota in 144 healthy infants over the first year of life, focusing on 92 infants from whom quantitative PCR- (qPCR) and NGS-based quantitative microbiota profiling data for absolute abundance estimation was available.^20^ This approach enables us to bypass the compositionality problem arising from relative abundances,^21^ which may mask true community dynamics,^14^ and lead to high false discovery rates.^20,22^ The bacterial abundances were further associated with parallelly collected data on gastrointestinal (GI) function, crying, and general health. We controlled for potential confounding factors using a carefully selected cohort, aiming to disentangle the effects of CS birth and IP antibiotics from potentially collinear effects.

## Results

### Study population

One hundred forty-four infants from the Finnish Health and Early Life Microbiota (HELMi)^23^ cohort and Jorvi cohort born by VD (N = 96) or CS (N = 48) were sampled during the first year of life for gut microbiota analysis. After the preprocessing and quality control of the 16S rRNA gene amplicon sequences, an average of 4.9 samples (range 1–7) was approved for analysis per infant (Table 1). Absolute bacterial profiles were calculated for a subset of 92 infants (VD: N = 46, CS: N = 46) with an average of 4.3 samples (range 1–6) per infant. The infants were categorized into five study groups based on birth mode and IP antibiotic (Table 1): (1) VD reference group without IP antibiotics, (2) exposure to cefuroxime or cephalexin in CS (CS-cep) or (3) exposure to cephalosporin in VD (VD-cep), (4) exposure to penicillin in VD (VD-pen), or (5) exposure to penicillin or any other antibiotics or a combination of antibiotics or the antibiotic class not reported in CS (CS-other). Due to low biomass in the samples from the first 2 d of life, 84% of the samples at day 1 and 50% at day 2 failed to reach adequate sequencing depth (120 reads, detailed in methods), from the whole dataset. The number of rejected samples did not differ statistically significantly by antibiotic exposure at either sampling time (χ^2^-test: day 1: *P* = 1 and day 2: *P* = .07, CS and VD IP antibiotic groups pooled).

**Table 1.** Fecal samples analyzed per delivery group and age (relative data/absolute data).

	VD	VD-cep	VD-pen	CS-cep	CS-Other
1 d	11/0	1/0	4/0	2/0	0/0
2 d	23/4	2/0	7/5	5/2	2/1
1 week	31/1	3/0	13/0	7/0	0/0
4 weeks	46/25	12/7	23/12	33/32	13/13
6 weeks	20/20	10/7	10/8	25/25	12/12
12 weeks	41/20	12/7	22/8	31/27	13/12
6 months	48/22	12/6	24/12	32/30	13/12
9 months	16/16	7/4	9/7	20/20	10/8
12 months	36/3	8/0	15/3	12/4	3/1

**Abbreviations**: VD, vaginal delivery; cep, cephalosporin; pen, penicillin; CS, Cesarean section.

IP antibiotics and doses varied among the delivery modes, with cefuroxime being the most common antibiotic used in CS and penicillin in VD (Table 2). Out of all the VD antibiotics, 74% were solely prophylactic, of which 93% to prevent GBS transmission and 7% due to premature rupture of membranes, while 69% of cephalosporins were therapeutic. Therapeutic IP antibiotics were administered due to fever or a rise in C-reactive protein (CRP) in the mother. Infant background statistics are summarized in Supplementary Table 1.

**Table 2.** Intrapartum antibiotics and doses by delivery group among the 144 infants including 92 with absolute data. The numbers of infants with relative data/absolute data are shown.

Birth Mode	Antibiotic	1 Dose	2 or More Doses	Total Count Of Infants
VD	Penicillin	4/1	21/12	25/13
	Cefuroxime	8/3	5/4	13/7
Elective CS	Cefuroxime	13/12		13/12
	Cephalexin	7/7		7/7
	Clindamycin	1/1		1/1
	Metronidazole	1/1		1/1
	Not reported	1/1		1/1
Emergency CS	Cefuroxime	10/10	1/1	11/11
	Cephalexin	3/3		3/3
	Clindamycin	2/2	2/2	4/4
	Penicillin		2/1	2/1
	Combination	1/1	4/4	5/5

**Abbreviations**: VD, vaginal delivery; CS, Cesarean section.

### The early development of the gut microbiota from birth to 12 months of age

Birth mode or antibiotic exposure did not influence the total bacterial abundance quantified by qPCR at any given sampling time (*P* > .05). Overall, the bacterial load increased with infant age in all groups, especially during the first weeks (Supplementary Figure 1).

**Figure 1.**
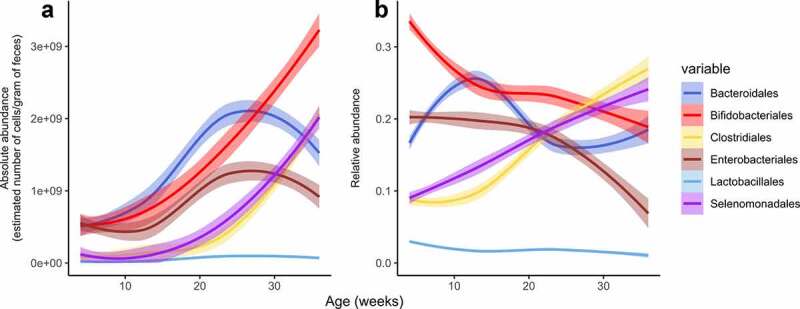
Temporal development of six most abundant bacterial classes in absolute (A, N = 26) and relative (B, N = 58) abundance in vaginally born infants not exposed to antibiotics. Smoothed curves are presented for samples collected between 4 weeks to 9 months. Absolute abundances are 16S rRNA gene copy number corrected, whereas relative abundances are not. The lines show means with 30% confidence interval.

To understand the temporal dynamics of the gut microbiota in infants, we analyzed the average developmental trajectory of the six most abundant bacterial classes in infants born with VD without antibiotics, comparing their relative and absolute abundances from all available data from 4 weeks to 9 months (Figure 1). The developmental trajectories of the major bacterial groups did not differ in relative abundance between the full dataset (N = 144) and the subset with absolute abundance data available (N = 92) (Supplementary Figure 2). Relative abundances show different community dynamics, such as a gradual decrease in the relative abundance of *Enterobacteriales* instead of a peak in absolute abundance between 6 and 9 months of age (Figure 1).

**Figure 2.**
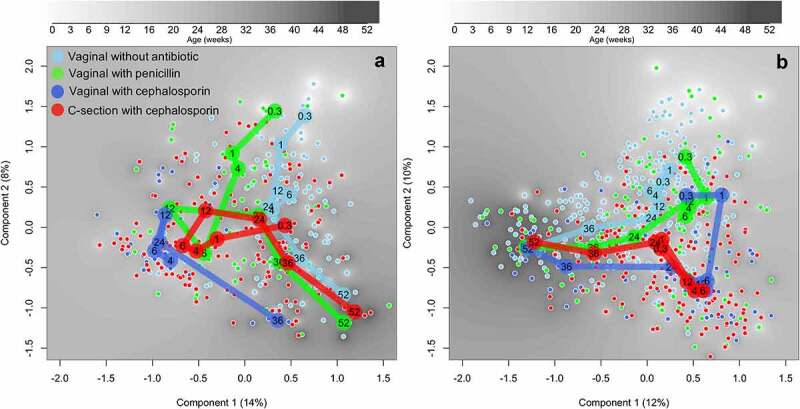
Principal coordinates analysis of the species-level absolute (A) and relative (B) abundances. Distances calculated based on Pearson correlations. The samples are colored by treatment group and the median component scores of the groups by age (weeks) presented by large circles.

The overall developmental trajectories of the microbiota differed between the study groups categorized by delivery mode and antibiotic type (Table 1) in the ordination space using principal coordinate analysis (PCoA) (Figure 2). The temporal development of the gut microbiota followed a discernible pattern in the reference group (VD without antibiotics), and each exposure group showed a distinct developmental trajectory based on both absolute (Figure 2a) and relative data (Figure 2b). At 2 d and 1 week, the groups did not differ significantly in their position in the principal component space, but by 4 weeks the differences reached statistical significance in both absolute and relative abundance data (*P* < .05). By 6 months (24 in Figure 2), all but VD-cep appeared to have converged with the reference group, but the differences were still statistically significant at 6–9 months (24–36 in Figure 2). By 12 months (52 in Figure 2), the differences in principal component space had disappeared (*P* > .05).

### Impact of birth mode and intrapartum antibiotic on the temporal development of the gut microbiota using absolute data

**Figure 3.**
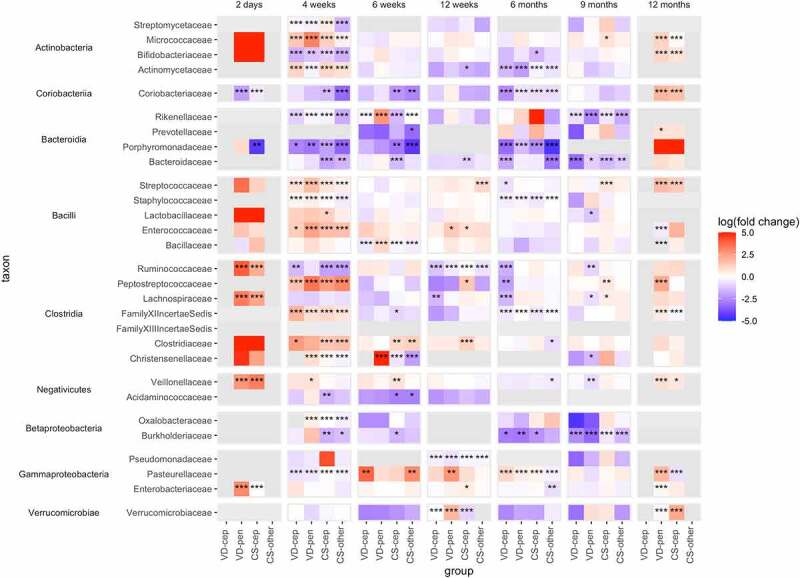
Fold changes calculated using absolute abundances of bacterial families with at least one group significantly different from the reference group (VD) in the taxon-wise comparisons. VD: vaginal delivery without antibiotic, VD-cep: vaginal delivery with cephalosporin, VD-pen: vaginal delivery with penicillin, CS-cep: C-section with cephalosporin, CS-other: C-section with any other antibiotic; fold change depicted logarithmic base 10, capped at 5 and −5 for clarity. *: P < .05, **: P < .01, ***: P < .001, in all cases FDR<0.1.

As the absolute abundance data better reflect the developing community dynamics,^14^ we focus on the absolute abundance data hereafter. We first compared abundances of bacterial families and genera of the exposure groups to the reference (VD without antibiotics), adjusting for breastfeeding, probiotics, and the introduction of solids when relevant. The results observed at the family level (Figure 3, Supplementary Figure 3) were largely consistent at the genus level (Supplementary Figure 4).

In the first 12 weeks, the absolute abundance of *Bacteroidaceae* was significantly reduced in the CS, but not in the antibiotic-VD groups (fold change range 0.010–0.11, *P* < .01 Figure 3). The depletion of *Bacteroidaceae* became statistically significant later, at 6 months and 9 months, in VD-cep (fold change 0.014, *P* = .0003) and in VD-pen (fold change 0.030, *P* = .01).

*Coriobacteriaceae* (Actinobacteria) followed a pattern similar to that of *Bacteroidaceae*, being mainly associated with birth mode. The absolute abundance of *Bifidobacteriaceae* was strongly and negatively affected by CS birth and by both cephalosporin and penicillin at 4 weeks (fold change range 0.0040–0.18, *P* < .01, Figure 3) when compared to VD without antibiotics. *Actinomycetaceae* and *Micrococcaceae* were enriched in all the exposure groups except the VD-pen group at 4 weeks (fold change range 6.2–13, *P* < .001, Figure 3). Families in the Bacilli class, *Staphylococcaceae, Enterococcaceae, Lactobacillaceae*, and *Streptococcaceae*, were increased in absolute abundance in all exposure groups during the first 12 weeks (fold change range 1.0–240, *P* < .05, Figure 3). The absolute abundances of several families in the classes Clostridia and Negativicutes were increased in all the exposure groups during the first month in comparison to the reference, with a few exceptions: reduced *Christensenellaceae* in the CS-other group at 4 weeks and in both CS groups at 6 weeks, reduced *Ruminococcaceae* in all but the VD-pen group at 4 and 12 weeks, and reduced *Acidaminococcaceae* in the CS groups at 4–6 weeks (Figure 3). The absolute abundances of Proteobacterial families in the antibiotic-exposed groups significantly deviated from the reference at multiple time points (fold change range 0.00054–6100, *P* < .05, Figure 3). Comparisons made using the relative data largely recapitulated the findings for prominently reduced taxa, such as *Bacteroidaceae* and *Bifidobacteriaceae* in the exposure groups, but lacked the majority of the significantly increased families (Supplementary Figure 5).

### Dissecting the effects of birth mode and intrapartum antibiotic on absolute levels of infant microbiota

We compared the birth modes while controlling for IP antibiotic exposure, including only cephalosporin-exposed CS and VD, to understand the effect of birth mode independently of antibiotic treatment, and *vice versa* (including only VD infants with different IP exposures) (Supplementary Figures 6, 7). Several bacterial families differed in absolute abundance between the VD-cep and CS-cep groups. In the CS-cep group, we observed enrichment of *Verrucomicrobiaceae* (3.8-fold difference, *P* < .001 at 6 weeks), *Bifidobacteriaceae* (18- and 12-fold differences, *P* < .05 at 4 and 12 weeks, respectively), *Streptomycetaceae* (7.0- and 62-fold differences, *P* < .001 at 4 weeks and 9 months), *Lactobacillaceae* (8.3- and 12-fold differences, *P* < .05 at 12 weeks and 9 months), *Bacillaceae* (3.8- and 8.5-fold differences, *P* < .001 at 6 weeks and 6 months), *Peptostreptococcaceae* (fold change range 7.5–5900, *P* < .01 at 4 and 12 weeks and 6 months), *Lachnospiraceae* (20- and 99-fold differences, *P* < .01 at 12 weeks and 6 months), *Clostridiaceae* (23-fold difference, *P* < .001 at 4 weeks), and *Christensenellaceae* (210-fold difference, *P* < .001 at 6 months). In contrast, *Bacteroidaceae* was reduced in CS-cep in comparison to VD-cep during weeks 4–12 (fold change 0.07, *P* < .01 at 6 weeks).

In terms of the effects of antibiotic types in the VD infants, increased absolute abundances were found in *Verrucomicrobiaceae* and *Rikenellaceae* (22- and 79-fold differences, *P* < .001 at 6 weeks), *Bifidobacteriaceae* and *Lactobacillaceae* (25- and 5.2-fold differences, *P* < .01 at 12 weeks), *Coriobacteriaceae* (fold change range 6.0–910, *P* < .001 at 4–12 weeks), *Peptostreptococcaceae* (94- and 72-fold differences, *P* < .05 at 4 weeks and 6 months), *Christensenellaceae* (8300-fold difference, *P* < .001 at 6 months), *Bacillaceae* (36- and 13-fold differences, *P* < .001 at 6 weeks and 6 months) in the VD-pen group compared to VD-cep. Members of *Clostridiaceae* were consistently reduced in VD-pen in comparison to VD-cep at 4 weeks (fold change 0.34, *P* < .001). Again, families significantly increased in the absolute comparisons were masked in the relative data comparisons (Supplementary Figure 8).

### Health outcomes

**Figure 4.**
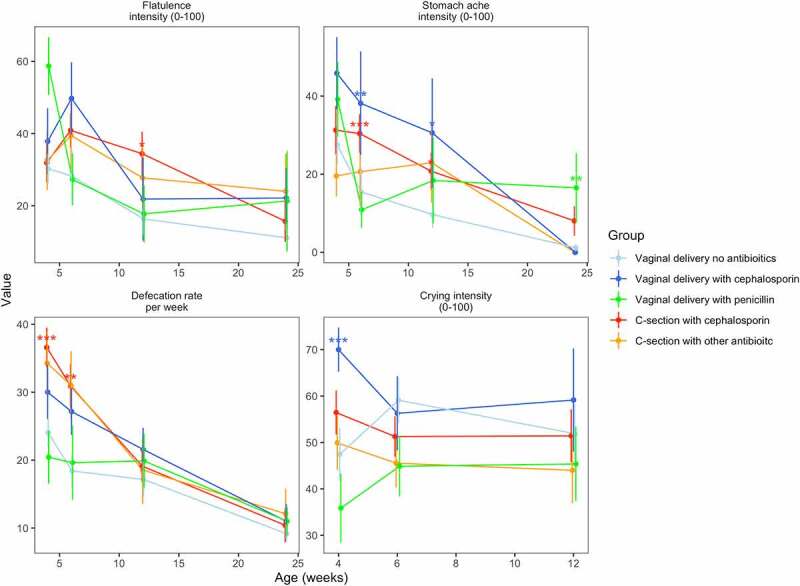
Parent-reported well-being outcomes at fecal sampling points from 1 to 6 by delivery group. *: P < .05, **: P < .01, ***: P < .001.

To determine whether the mode of delivery and IP antibiotics were associated with the infants’ well-being during the first year of life, we compared the study groups in terms of parent-reported digestive and overall health from 73 infants with absolute microbiota profiles and health variables available (Supplementary Table 1). Infants’ overall health as reported by the parents did not differ between the study groups at any sampling age (ANOVA: *P* > .1) nor did the cumulative number of antibiotic courses by 12 months (*P* = .81) nor infant weights at birth or at 12 months (ANOVA: *P* ≥ .17, Supplementary Table 2). All exposure groups showed increased GI symptoms mainly during the first 12 weeks compared to the reference group VD. There were significant differences in defecation rate, crying intensity, parent-perceived stomach pain intensity, and flatulence between the study groups (Figure 4). We assessed the hypothesis that differences in the gut microbiota mediate GI symptoms and crying by analyzing the association between antibiotic exposure and birth mode as separate variables with the well-being outcomes and identified bacterial taxa associated with both the outcomes and the delivery antibiotic exposure or birth mode. Results are detailed below by age.

#### Week 4

**Figure 5.**
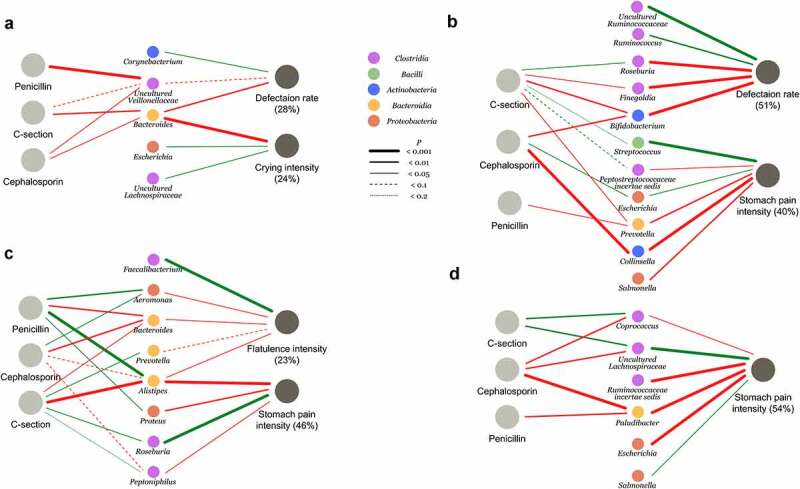
Associations between birth interventions, absolute abundances of bacteria, and GI symptoms at 4 weeks (A), 6 weeks (B), 12 weeks (C) and 6 months (D). The amount of variation explained by the bacteria shown is given as percentage. The models were selected based on Akaike information criterion (AIC). In some cases, the final model retained variables that were not statistically significant but contributed significantly to the model.

At 4 weeks, cephalosporin exposure (including both VD and CS) was significant and positively associated with crying intensity (*P* = .009). A model using absolute bacterial abundances explained 24% of the variation in crying intensity (Figure 5aA). The association of cephalosporin exposure with crying intensity appeared to be mediated by the low abundance of *Bacteroides*. Defecation rate was positively associated with cephalosporin exposure (*P* < .0001) and CS (*P* < .0001). The bacterial composition explained 28% of the variation in defecation rate. The increased defecation rate in CS-born and cephalosporin-exposed infants compared to the controls appeared attributable to the decreased abundances of *Bacteroides* and uncultured *Veillonellaceae* (Figure 5a).

#### Week 6

At 6 weeks, both CS birth (*P* = .006) and cephalosporin exposure (*P* = .01) were independently associated with increased defecation rate. The gut microbiota composition was associated with the defecation rate, explaining 51% of the variation. The effects of CS birth and cephalosporin appeared to be mediated by the decreased abundances of *Bifidobacterium* and *Finegoldia* (Figure 5B). Cephalosporin exposure was significantly associated with the parent's perceived stomach pain (*P* = .005). The association between cephalosporin exposure and stomach pain was mediated by the reduced abundance of *Collinsella* and the increased abundances of *Escherichia* and *Streptococcus* in the CS-born and cep-exposed infants (including VD-cep) (Figure 5B). Gut microbiota composition explained 30% of the variation in stomach pain intensity.

#### Week 12

At 12 weeks, cephalosporin exposure was significantly associated with the stomach pain intensity (*P* = .02). The gut microbiota explained 46% of variation in stomach pain, low absolute abundance of *Alistipes*, and high abundance of *Roseburia* being linked with both CS birth and stomach pain (Figure 5c). CS birth (*P* = .05) and cephalosporin exposure (including CS-cep and VD-cep) (*P* = .02) were associated with increased flatulence intensity. The gut microbiota explained 23% of the variation in flatulence intensity at 12 weeks. The association of CS birth with cephalosporin and flatulence appeared mediated by the reduced abundance of *Bacteroides* and *Alistipes* (Figure 5C).

#### Month 6

At 6 months, stomach pain intensity was associated with penicillin exposure (*P* = .01). Gut microbiota composition explained 54% of the variation in stomach pain intensity, with *Paludibacter* mediating the association with penicillin exposure (Figure 5D).

## Discussion

We set out to study how temporal quantitative and compositional gut microbiota development is influenced by the mode of delivery versus exposure to delivery antibiotics, and whether these early exposures and their associated differences in the early gut microbiota relate to common gastrointestinal symptoms in infants. In addition to the profound effects of CS delivery on the gut microbiota, we observed that maternal IP antibiotics compromised the colonization of the newborn’s gut microbiota in VDs. Notably, the decreased absolute abundance of *Bifidobacterium* and increased abundance of Bacilli characterized both CS-deliveries and IP antibiotic-exposed VD infants, whereas a reduction in *Bacteroides* was mainly seen after CS delivery. Changes in gut microbiota related to CS birth and IP antibiotic exposure were further associated with increased GI symptoms.

To the best of our knowledge, this is the first study using quantitative microbiota profiling for community-wide absolute abundance estimates of infants’ gut bacteria,^20,24^ and the first to address the associated GI symptoms. The preponderance of existing NGS studies in microbiota research is solely based on relative abundance data. This has been recently shown to under- or overestimate temporal changes of the gut microbiota in preterm infants,^14^ especially concerning Proteobacteria such as *Escherichia coli* and *Klebsiella*. In the present study, we documented a decreasing developmental trajectory of the most abundant Proteobacterial order *Enterobacteriales* in relative abundance data, in line with earlier studies based on relative abundance.^25^ In contrast, the absolute abundance of *Enterobacteriales* increased over the first 6 months of life, in agreement with previous studies in term infants utilizing targeted qPCR.^26,27^ The developmental patterns of other dominant bacteria were largely in concordance with the previous qPCR-based study, showing a general upward trend in the first few months of life.^26^ Relative abundances were also underestimated for taxa with low 16S rRNA gene copy numbers, such as *Bifidobacteriales* (mean 3.5 copies^28^), compared to taxa with high copy numbers, such as *Clostridiales* (mean 5.5 copies^28^), as observed in this study. Quantitative microbiota profiling captures inter-personal temporal variability and ecosystem maturation in the gut microbiota better than relative microbiota profiling.^29^ This inter-personal variability and distinct developmental trajectories of several bacterial taxa captured only in absolute abundance were reflected in the mediation analysis, where some significant associations were not consistent throughout all sampling points.

Although the common belief is that CS birth causes microbial deprivation, the total bacterial loads did not differ between CS-born, antibiotic-exposed, and non-exposed VD infants 4 weeks onwards in our study. We observed a clear age-driven increase in the bacterial loads in all study groups. The possibly transient difference in total microbial load before 4 weeks of age could not be evaluated in the present study due to the low bacterial biomass samples. Previous studies have reported lower bacterial loads in CS-born infants,^30^ and a faster age-driven increase in bacterial quantities in the stools of CS-born infants.^31^

Studies relying on relative abundance have consistently documented an altered bacterial signature in the gut microbiota of CS-born infants,^1,32,33^ recently shown to be restored by maternal FMT.^34^ Most studies on infant microbiota in relation to birth mode have neglected the fact that practically all CS births involve exposure to IP antibiotics, rendering it impossible to isolate the effects of IP from the birth route.^35^ The administration of IP antibiotics is known to alter the development of VD infants’ gut microbiota.^10,36^ Our results comparing the effect of birth mode under the exposure to the same antibiotic, cephalosporin, confirmed that birth mode *per se* influences the infant gut microbiota. These results are corroborated by two recent randomized trials that documented a lack of clear differences in the gut microbiota of CS-born infants exposed versus unexposed to IP antibiotic.^37,38^ In conclusion, our study and earlier data indicate that the mode of birth is a major factor affecting neonatal colonization, in line with CS, which eliminates the contact between the infant and maternal gut bacteria, preventing their transmission at birth.^39^

In contrast, in VD infants the IP antibiotic plays a major role. We showed that especially the impact of cephalosporin exposure during VD was largely similar to the effect of CS delivery on the infant gut microbiota. However, we identified several distinct effects of birth mode and antibiotic exposure. Namely, the absolute abundances of *Bacteroidaceae, Coriobacteriaceae*, and *Burkholderiaceae* were reduced by CS birth but not by IP antibiotics during first 6 weeks, while *Ruminococcaceae, Porphyromonadaceae, Rikenellaceae*, and *Pasteurellaceae* abundances were negatively affected by both CS birth and antibiotics. *Bifidobacteriaceae* was most strongly affected by antibiotic exposure. Our observations recapitulate and extend the results of recent studies.^36,40,41^ Using absolute abundance data, we found the effect of IP antibiotics in VD infants to peak at 12 weeks with some differences that persist up to 12 months. Previous studies using relative abundance data have reported that the effect of birth-related antibiotics typically subsides after 3 months;^42^ however, most of the studies have not investigated the effect beyond this age.^12^ A recent prospective study of 100 VD infants reported that the effects of IP antibiotics overruled those of postnatal antibiotics on infant microbiota and persisted until the age of 1 y.^43^

Importantly, several bacterial taxa were increased in absolute abundance in the CS-born and in the antibiotic-exposed infants. For example, IP antibiotic exposure in VD and CS births both resulted in an increase in streptococci, as well as staphylococci and enterococci. This is likely due to reduced colonization resistance resulting from the depletion of bifidobacteria, which have anti-streptococcal activity.^44^ In fact, the majority of the bacterial taxa affected by IP antibiotics, except bifidobacteria, was increased in absolute abundance during the first month. This suggests that the major driver of microbial changes after exposure to IP antibiotics is not the antibiotic itself but the antibiotic-induced reduction in bifidobacteria. Since the direct effects of IP antibiotics on infants are short (from hours to a couple of days), the lasting effects likely reflect the consequences of fundamental alterations in ecosystem assembly.^45^ The pattern is somewhat different after CS birth, where diverse members of the microbiota, including *Bacteroides*, bifidobacteria, and members of Clostridiales and Negativicutes, were likely directly reduced due to lower mother–infant microbiota transmission rates during CS birth.^6^

To our knowledge, our study is the first to evaluate the influence of birth mode and associated maternal antibiotic administration in parallel on the infant gut microbiota and functional GI symptoms. The CS birth and postnatal antibiotics are known risk factors for functional GI disorders in pre- and full-term infants.^46^ In our study, CS-born and VD IP antibiotic-exposed infants experienced more parent-reported abdominal discomfort and higher intensity of crying compared to VD infants with no IP antibiotic exposure, while their general health and growth during the first year was comparable. Previously, analogous mediation analysis has been used *e.g*. to study the role of infant gut microbiota in ethnicity-associated development of food sensitization.^47^ Colic symptoms have previously been linked with the reduced relative abundance of bifidobacteria,^48,49^ taxon also found in our associations, and to an altered balance between H_2_-producing and -utilizing taxa.^50^ Overall, the models based on absolute bacterial abundances explained 23–54% of the infants’ GI symptom variation, which is remarkable considering that the origin and underlying reasons of functional GI symptoms in infants often remain unclear.^51^

*Bacteroides* and bifidobacteria, associated with infant well-being in the current study, are the major human milk oligosaccharide (HMO)-degraders and thus keystone taxa in the breastfed infant gut,^52–54^ so their depletion will have profound impact on metabolic processes, pH, and other conditions^55^ in the infant gut.^56^ Such changes likely affect gut physiology and function and may favor bacteria that are normally less abundant in the infant gut, such as members of Clostridia, which in the present study were associated with increased defecation rates, flatulence, and stomach pain.^7,8,18^ Overall, our results on health associations are preliminary and derived from a relatively small study, and hence need validation in future cohorts.

Together, CS and antibiotic exposure at birth affect a substantial proportion of infants worldwide. Our data suggest that their effects on early colonization of the gut microbiota may have reduced the well-being of infants during the first months of life, but reassuringly their overall health was comparable to that of non-exposed infants. This study is an important primer for filling the knowledge gaps that underlie the lack of consensus for the choice between universal or risk-based screening for IP antibiotic use^9^ and contributes to weighing the benefits and potential harms of antibiotic use in mothers and infants.

## Materials and Methods

### Study design

Infants specifically recruited for this study (Jorvi cohort, N = 68) and additional 83 infants recruited for the HELMi cohort^23^ (NCT03996304) were included in the study. In both cohorts, pregnant women with singleton gestation were recruited from the general population in Southern Finland. Healthy babies, born in gestational weeks 37–42, without known congenital defects and exceeding the birth weight of 2.5 kg were included in the study (except for one baby with a birth weight of 2.4 kg). At least one parent had to be Finnish speaking to be able to answer the questionnaires.

For the Jorvi cohort, 31 mothers delivered with (VD N = 26/31) and 37 without IP antibiotics (all VD). Infant fecal samples were collected on days 1 and 2, at 1, 4, and 12 weeks, and 6 and 12 months. From the HELMi cohort, 83 infants were selected based on their birth mode, IP antibiotics, and the availability of sequencing data from infant’s fecal samples at the age of 3 (considered as week 4 sample), 6, and 12 weeks, and 6, 9, and 12 months. From the combined cohort, five infants were excluded as they received post-natal antibiotics prior to the first sampling point, and two infants were excluded for low sequence quality, leaving a total of 144 infants. Additionally, all samples following a post-natal antibiotic treatment were excluded from the analysis as well as individual samples with low sequence quality (see Statistical analysis). We divided the remaining infants into five study groups based on birth mode and IP antibiotics: VD reference group without IP antibiotics (N = 58), CS-cep (N = 34), VD-cep (N = 13), VD-pen (N = 25), and CS-other (N = 14) (Table 1). The CS groups comprised elective (N = 23) and emergency (N = 25) cases and all involved IP antibiotics and were pooled for the analyses (Supplementary results: Supplementary Figure 9). Based on sample sufficiency, birth mode, and sampling point, the samples from 92 infants were quantified for total bacteria by qPCR,^20^ representing 26 infants in the reference group VD, 7 in VD-cep, 13 in VD pen, 33 in CS-cep, and 13 in CS-other group.

Both cohort studies were approved by the ethical committee of the Hospital District of Helsinki and Uusimaa and performed in accordance with the principles of the Helsinki Declaration. Parents signed an informed consent at enrollment.

### Samples and data collection

Parents collected fecal samples from the infants and stored them at −20°C. The samples were transported frozen to the study center within 6 months for −80°C storage. Data on feeding regime, probiotic and antibiotic uses, and child health and growth were collected with questionnaires (HELMi) or via phone interviews from the parents at the time of stool sampling (Jorvi). Data on exposures, *i.e*., the mode of delivery and the usage, type, and dosing of maternal IP antibiotics were collected from hospital records for both cohorts.

### Health outcomes

Infant health and well-being were addressed using questionnaire data available for the HELMi cohort.^23^ We used data from weekly to monthly repeating questionnaires using the questionnaire closest to each fecal sampling. The variables included were related to crying intensity estimated by a 0–100 mm visual analog scale (VAS), collected until 6 months and GI function (defecation rate, parent-perceived stomach pain intensity, and flatulence) until 9 months. We also compared the frequency of infections estimated by the number of postnatal antibiotic courses by the age of 12 months. Infant growth was measured in postnatal care visits and transformed into age-dependent WHO z-scores.^57^

### Microbiota profiling

Bacterial DNA was extracted from the fecal samples using a previously described bead beating method,^58^ and KingFisherTM Flex-automated purification system (ThermoFisher Scientific). The fecal DNA was extracted from 250 to 340 mg of fecal material that was suspended in 0.5–1 ml of sterile ice-cold PBS, and 175–250 μl of fecal suspensions was combined with 235–250 μl of RBB lysis buffer (500 mM NaCl, 50 mM Tris-HCl (pH 8.0), 50 mM EDTA, 4% SDS) in a bead-beating tube from the Ambion MagMAX™ Total Nucleic Acid Isolation Kit (Life Technologies). After repeated bead-beating, 200 μl of the supernatant was used for DNA extraction with a KingFisher^TM^ Flex automated purification system (ThermoFisher Scientific) using a MagMAX^TM^ Pathogen High Vol. DNA was quantified using Quanti-iT™ Pico Green dsDNA Assay (Invitrogen).

The 16S rRNA gene amplicon sequencing was performed using Illumina MiSeq and HiSeq platforms for V3-V4 or V3 region as previously described,^20,59^ respectively. The two sequencing platforms give comparable results (Supplementary Figure 10). Due to the low DNA and/or read yields, for 161 samples the V3-V4 library preparation protocol was modified with one or more of the following modifications: additional DNA concentration with ethanol precipitation, 2-step index PCR, increased input of template DNA (5 vs 1 ng/rx), and more PCR cycles (45 vs 27).

The numeration of total bacteria was performed in triplicate as previously described.^20^ Bacterial DNA was quantified by amplifying 0.5 ng aliquots of each DNA extract, with universal bacterial primers 331 F/797R^60^ targeting the 16S rRNA gene. Standard curves ranging from 10^2^ to 10^7^ copies were constructed using the full-length amplicons of 16S rRNA gene of *Bifidobacterium bifidum* to convert the threshold cycle (Ct) values into 16S rRNA gene copy numbers per g of feces.

The sequencing reads were processed using R package mare,^61^ which relies on USEARCH^62^ for quality filtering, chimera detection, and taxonomic annotation. Only the forward reads (V3), truncated to 150 bases, were used.^63^ Reads occurring <10–50 times, depending on run size, were excluded as potentially erroneous. The taxonomic annotation was performed using USEARCH^62^ by mapping the reads to the SILVA 16S rRNA reference database version 115,^64^ restricted to gut-associated taxa. Further, potential contaminants in the low-DNA-yield samples were filtered by removing reads appearing in negative controls (PCR or extraction blanks) in corresponding numbers from all samples. The absolute abundances were estimated and 16S rRNA gene copy-number corrected as previously described by dividing the absolute abundances with taxon-specific copy numbers from the rrnDB^28^ database.^20^

### Statistical analysis

After processing, the median number of reads obtained was 26 384 (range 3 to 253 458). Sufficient sequencing coverage was evaluated by comparing observed species richness to read counts (Supplementary Figures 11 and 12) and a cutoff of 2000 reads was chosen for HELMi samples, and 120 for Jorvi samples (to accommodate low-biomass samples from the first week of life). Samples with insufficient coverage (N = 138, largely samples from the first week of life) and 69 samples with over 60 000 reads from a single HiSeq run due to high amplification of probable contaminants (mostly *Alcaligenaceae*)^65^ were excluded, leaving a total of 688 high-quality samples from 144 infants for downstream analyses, and 393 high-quality qPCR quantified samples from 92 infants.

The statistical analysis was conducted in R version 3.6.3 with the package *mare*,^61^ with tools from packages *vegan*,^66^ *MASS*,^67^ and *nlme*.^68^ The effects of birth mode and IP antibiotics on the abundances of the bacterial taxa were analyzed using, primarily, negative binomial, secondarily, Poisson, or tertiarily, quasi-Poisson models, depending on the data distribution and model fit, using the VD group without antibiotics as the reference group. If the fitted model failed to fulfill model assumptions (primarily heteroscedasticity of the residuals), generalized least-squares models were used. Only the genera observed in >30% of the samples were analyzed individually. To account for multiple comparisons, only results with false discovery rate (FDR) values <0.1 were considered significant. All models were adjusted for infant probiotic intake at the time of sampling (none, *Lactobacillus* spp., *Bifidobacterium* spp. or both, or *Saccharomyces* spp.), feeding type, including breastfeeding status (none, partial, exclusive) and weeks since the introduction of solids, and the sample treatment history, including possible PCR modifications and sequencing run ID. Principal coordinate analysis was done by log-transforming the data and calculating Pearson correlation-based distances between samples. Analysis was done using the capscale function in the R package vegan. The significance of the group differences at each time point in the first two principal coordinates was analyzed using ANOVA.

The health outcome variables were compared between the birth groups at each fecal sampling until 9 months using a negative binomial model adjusted for feeding type and probiotic use. Additionally, the association of antibiotic exposure (no antibiotic, penicillin, and cephalosporin) and birth mode (VD or CS) with health outcomes was analyzed at each fecal sampling until 9 months using a negative binomial model adjusted for feeding type and probiotic use. To determine if differences in infant well-being were mediated by changes in gut microbiota and the associated genera, the *PathModel* function in *mare*^61^ was used to identify taxa predicting symptoms and the role of birth interventions related to the predictive taxa using absolute bacterial counts.

## Supplementary Material

Supplemental Material
