# Supplementary material for: Quantitative insights into effects of intrapartum antibiotics and birth mode on infant gut microbiota in relation to well-being during the first year of life
Source: Gut Microbes. 2022 Sep 29;14(1):2095775. doi: 10.1080/19490976.2022.2095775 (PMC9542534; doi:10.1080/19490976.2022.2095775)
Supplement: Supplemental Material [file KGMI_A_2095775_SM1219.zip › Supplemental_materials_Gut_Microbiomes_revised.docx]

# Supplemental material

## Supplementary Tables

Supplementary Table 1 Group sizes for gastrointestinal symptom mediation analysis.

| **Age** | **VD** | **VD-cep** | **VD-pen** | **CS-cep** | **CS-other** | **Total** |
| --- | --- | --- | --- | --- | --- | --- |
| 4 weeks | 21 | 7 | 7 | 27 | 11 | 73 |
| 6 weeks | 20 | 7 | 8 | 25 | 11 | 71 |
| 12 weeks | 20 | 7 | 8 | 26 | 11 | 72 |
| 6 months | 19 | 6 | 6 | 22 | 10 | 63 |
| 9 months | 16 | 4 | 6 | 20 | 7 | 53 |

VD = vaginal delivery, CS = Caesarean section, cep = cephalosporin, pen = penicillin

Supplementary Table 2 Infant background variables summarized by study group.

|  |  | **VD** | **VD-cep** | **VD-pen** | **CS-cep** | **CS-other** |
| --- | --- | --- | --- | --- | --- | --- |
| **Male (N=72, 50%)** | **Average birth weight (kg)** | 3.56  SD=0.47  N=26 | 3.50  SD=0.46  N=8 | 3.36  SD=0.50  N=12 | 3.62  SD=0.42  N=17 | 3.71 SD=0.61  N=9 |
|  | **Average weight at 1 year (kg)** | 9.54  SD=0.88  N=24 | 10.7  SD=1.63  N=4 | 9.63  SD=0.97  N=8 | 9.74  SD=0.69  N=15 | 10.1 SD=0.72  N=9 |
| **Female (N=72, 50%)** | **Average birth weight (kg)** | 3.49  SD=0.39N=32 | 3.53  SD=0.24N=5 | 3.37  SD=0.45N=13 | 3.42  SD=0.42N=17 | 3.64 SD=0.56 N=5 |
|  | **Average weight at 1 year (kg)** | 9.39  SD=0.97  N=24 | 9.42  SD=0.87  N=5 | 9.08  SD=0.94  N=10 | 9.32  SD=1.4  N=13 | 9.91  SD=1.6  N=5 |
| **Breast-feeding** | **Exclusive**  **at 3 months** | 80.5% | 91.7% | 54.6% | 77.4% | 76.9% |
|  | **Any**  **3 months** | 95.1% | 100% | 100% | 93.6% | 100% |
|  | **Any**  **6 months** | 91.7% | 91.7% | 100% | 90.6% | 92.3% |
|  | **Any**  **9 months** | 93.8% | 85.7% | 88.9% | 80.0% | 80.0% |
|  | **Any**  **12 months** | 72.2% | 62.5% | 60.0% | 75.0% | 66.7% |
| **Solid foods** | **Age of introduction (months)** | 4.63 SD=0.94 | 4.69 SD=0.85 | 4.24  SD=0.52 | 4.71 SD=0.87 | 4.79 SD=0.89 |
| **Pro-biotic intake** | **During 1^st^ year** | 94.8% | 53.5% | 72.0% | 85.3% | 57.1% |

VD = vaginal delivery, CS = Caesarean section, cep = cephalosporin, pen = penicillin, SD = standard deviation

## Supplementary Figures

Supplementary Figure 1 Logarithmically-transformed total bacterial counts at different age grouped by delivery group. No significant difference between the bacterial loads (*P<*0.5) was found between the reference group (VD no antibiotics) and any of the exposure groups at any given age.

Supplementary Figure 2 Temporal development of six most abundant bacterial classes in relative abundance in the full dataset (upper panel; N=144) and the subset with available data for quantitative microbiota profiling (lower panel; N=92) by different intrapartum antibiotic exposure groups. No apparent differences between the two datasets can be found.

Supplementary Figure 3 Boxplots depicting the absolute abundances of selected bacterial families significantly differing from the reference group (VD) in the taxon-wise comparisions. Models used to calculate the different abundances included feeding, probiotics and technical confounding. *: P<0.05, **: P<0.01, ***: P<0.001, in all cases FDR<0.1.

Supplementary Figure 4 Fold changes calculated using absolute abundances of bacterial genera with at least one group significantly differing from the reference group (VD) in the taxon-wise comparisons. VD: vaginal delivery without antibiotic, VD-cep: vaginal delivery with cephalosporin, VD-pen: vaginal with penicillin, CS-cep: C-section with cephalosporin, CS-other: C-section with any other antibiotic; fold change depicted logarithmic base 10, capped at 5 and -5 for clarity; *: P<0.05, **: P<0.01, ***: P<0.001, FDR<0.1.

Supplementary Figure 5 Fold changes calculated using relative abundances of bacterial families with at least one group significantly differing from the reference group (VD) in the taxon-wise comparisons. VD: vaginal delivery without antibiotic, VD-cep: vaginal delivery with cephalosporin, VD-pen: vaginal with penicillin, CS-cep: C-section with cephalosporin, CS-other: C-section with any other antibiotic; fold change depicted logarithmic base 10, capped at 5 and -5 for clarity; *: P<0.05, **: P<0.01, ***: P<0.001, FDR<0.1.

Supplementary Figure 6 Fold changes calculated using absolute abundances of bacterial families with at least one group significantly differing from the VD-cep group in the taxon-wise comparisons. VD-cep: vaginal delivery with cephalosporin, VD-pen: vaginal with penicillin, CS-cep: C-section with cephalosporin; fold change depicted logarithmic base 10, capped at 5 and -5 for clarity; *: P<0.05, **: P<0.01, ***: P<0.001, FDR<0.1.

Supplementary Figure 7 Fold changes calculated using absolute abundances of bacterial genera with at least one group significantly differing from the VD-cep group in the taxon-wise comparisons. VD-cep: vaginal delivery with cephalosporin, VD-pen: vaginal with penicillin, CS-cep: C-section with cephalosporin; fold change depicted logarithmic base 10, capped at 5 and -5 for clarity; *: P<0.05, **: P<0.01, ***: P<0.001, FDR<0.1.

Supplementary Figure 8 Fold changes calculated using relative abundances of bacterial families with at least one group significantly differing from the VD-cep group in the taxon-wise comparisons. VD-cep: vaginal delivery with cephalosporin, VD-pen: vaginal with penicillin, CS-cep: C-section with cephalosporin; fold change depicted logarithmic base 10, capped at 5 and -5 for clarity; *: P<0.05, **: P<0.01, ***: P<0.001, FDR<0.1.

Supplementary Figure 9 Differences in bacterial families between the two C-section types.

Supplementary Figure 10. Bacterial community composition of mock communities 3 and 4 (MC3 and MC4) by Ramiro-Garcia et al. (DOI: [10.12688/f1000research.9227.2](https://doi.org/10.12688/f1000research.9227.2)) sequenced by MiSeq (M) and HiSeq (H) compared to the reference (R) composition.

Supplementary Figure 11 OTU-richness plotted in regards of the natural logarithm of sample read count of the HELMi cohort sample (N=524). The vertical line marks the read count cut-off value of 2000 reads.

Supplementary Figure 12 OTU-richness plotted in regards of the natural logarithm of sample read count of the Jorvi cohort sample (N=644). The vertical line marks the read count cut-off value of 120 reads.

## Supplementary results

We tested whether the type of CS (elective versus emergency) was associated with the gut microbiota composition in infants before pooling the different CS types. The relative abundance of *Bacteroides* was reduced in elective compared to emergency CS at week 1, 3 and 6 (3.6-8.9-fold difference, *P*<0.005), and 6 months (5.1-fold difference, *P*=0.02). The difference in absolute abundance was not significant. *Bifidobacterium* was significantly reduced at 6 months in elective CS vs emergency in both relative abundance (9.8-fold difference, *P*=0.002) and absolute abundance (5.7-fold difference, *P*=0.03). Although there were subtle differences between the microbiota after emergency and elective CS (Supplementary Figure 1), the differences between the microbiota of CS versus vaginally-delivered infants (independent of IP antibiotics) remained large even after pooling, justifying the grouping of CS types (Table 1).
