# Supplementary figures and images for: Quantitative insights into effects of intrapartum antibiotics and birth mode on infant gut microbiota in relation to well-being during the first year of life
Source: Gut Microbes. 2022 Sep 29;14(1):2095775. doi: 10.1080/19490976.2022.2095775 (PMC9542534; doi:10.1080/19490976.2022.2095775)

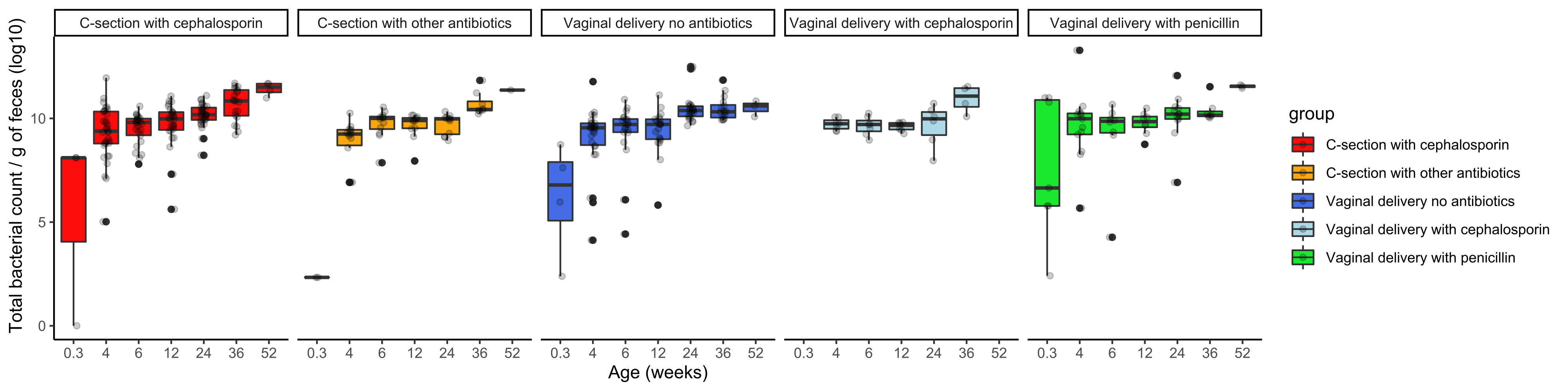

Supplement: Supplemental Material [file KGMI_A_2095775_SM1219.zip › Supplementary_figure1.tiff]

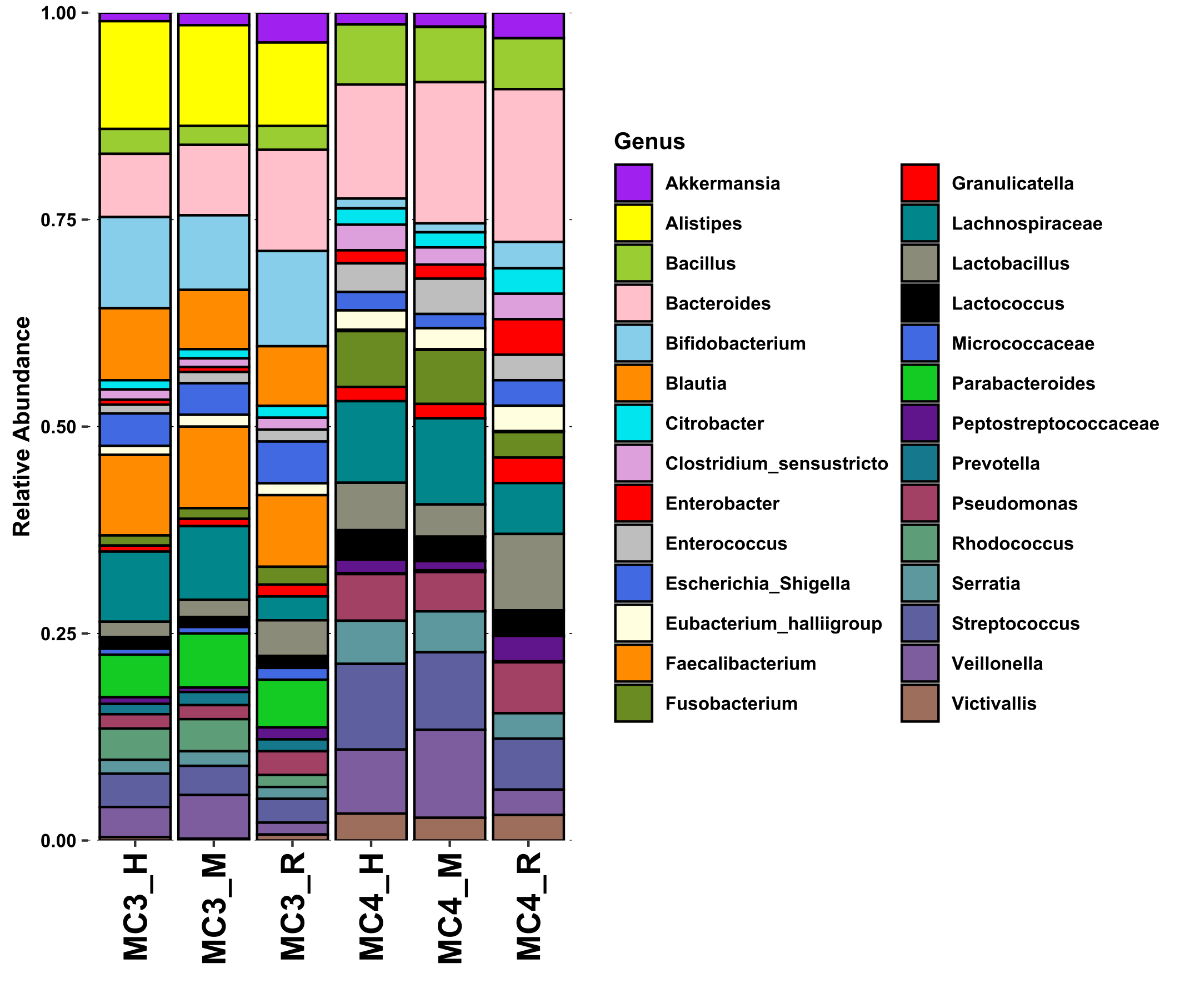

Supplement: Supplemental Material [file KGMI_A_2095775_SM1219.zip › Supplementary_figure10.tiff]

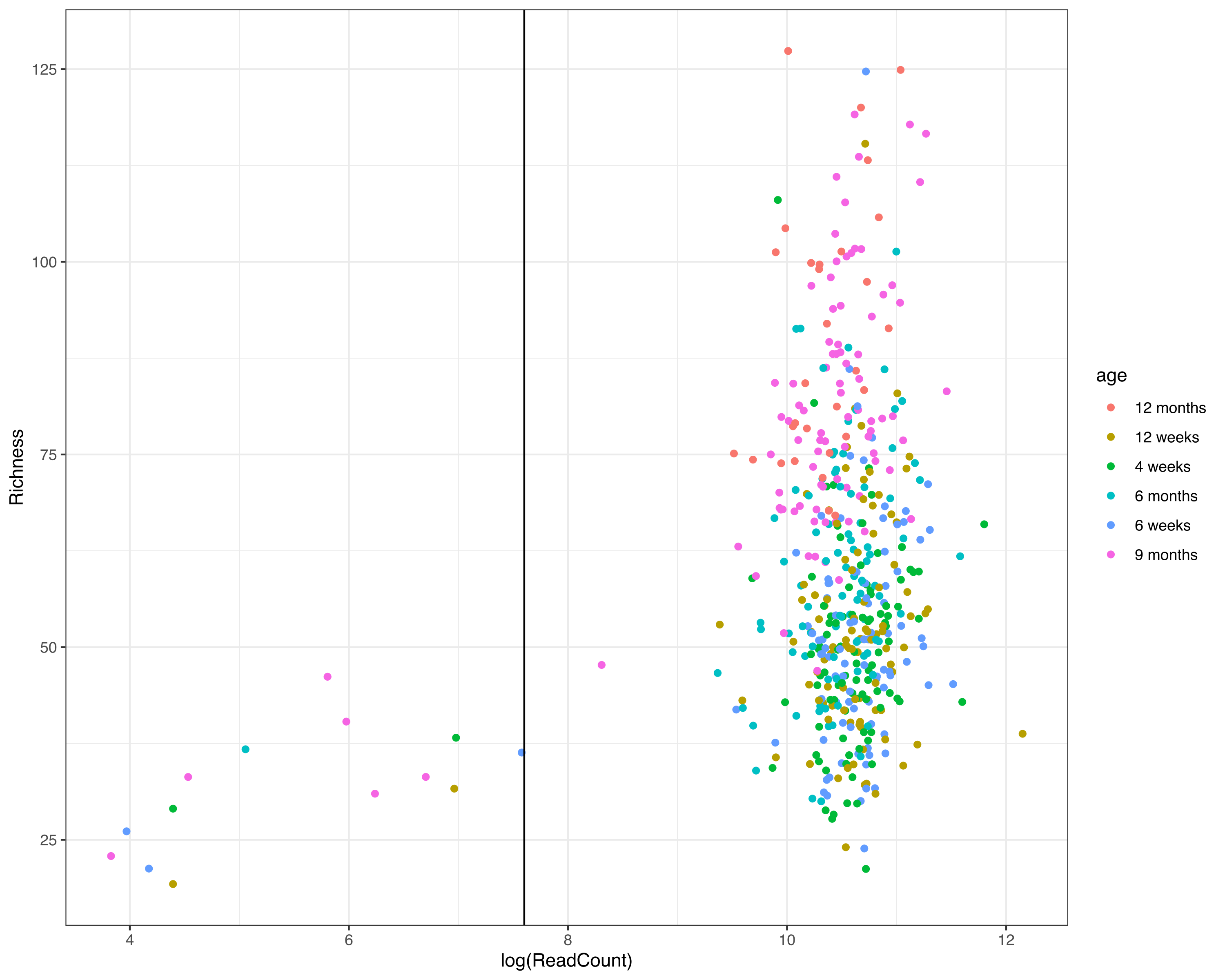

Supplement: Supplemental Material [file KGMI_A_2095775_SM1219.zip › Supplementary_figure11.tiff]

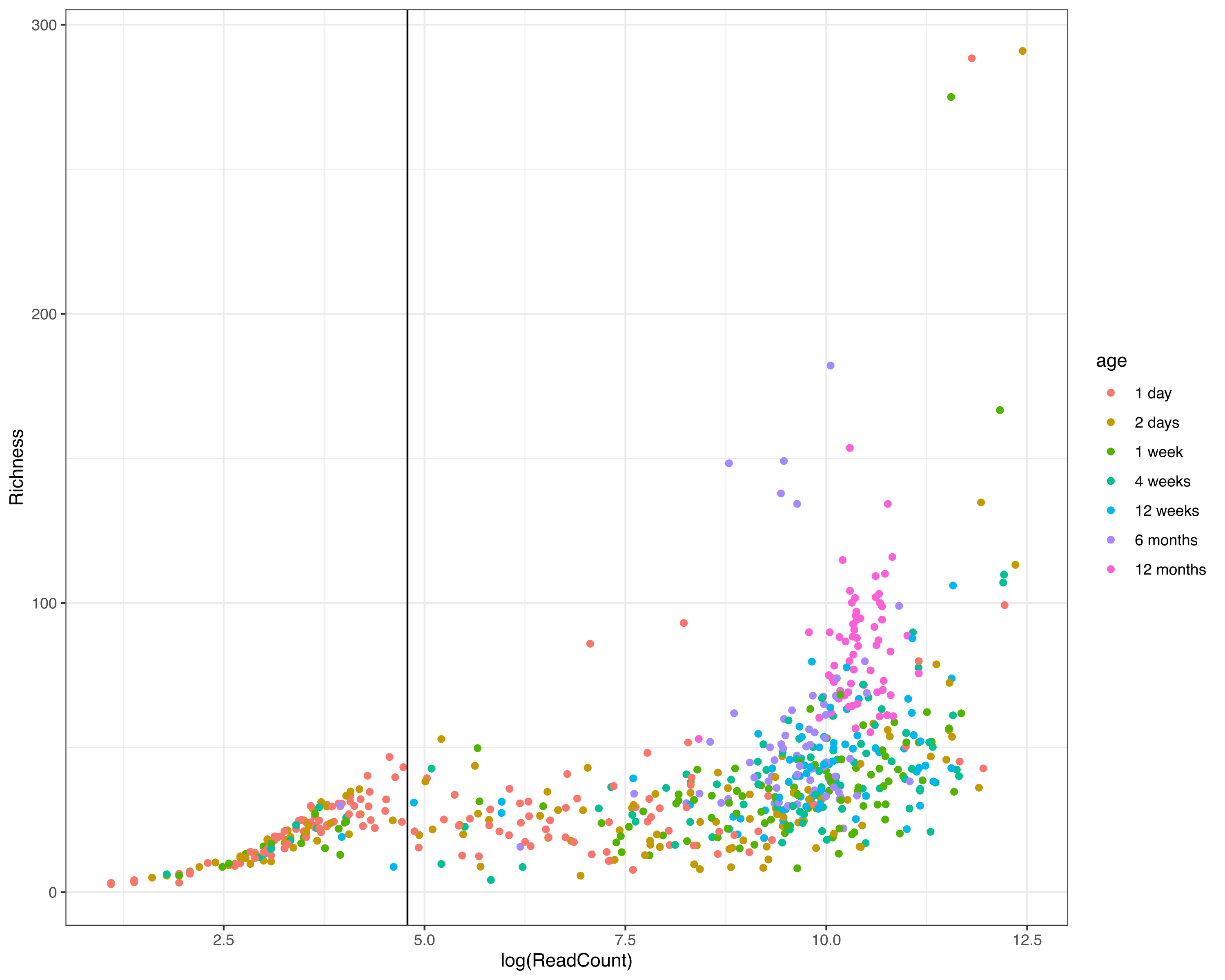

Supplement: Supplemental Material [file KGMI_A_2095775_SM1219.zip › Supplementary_figure12.tiff]

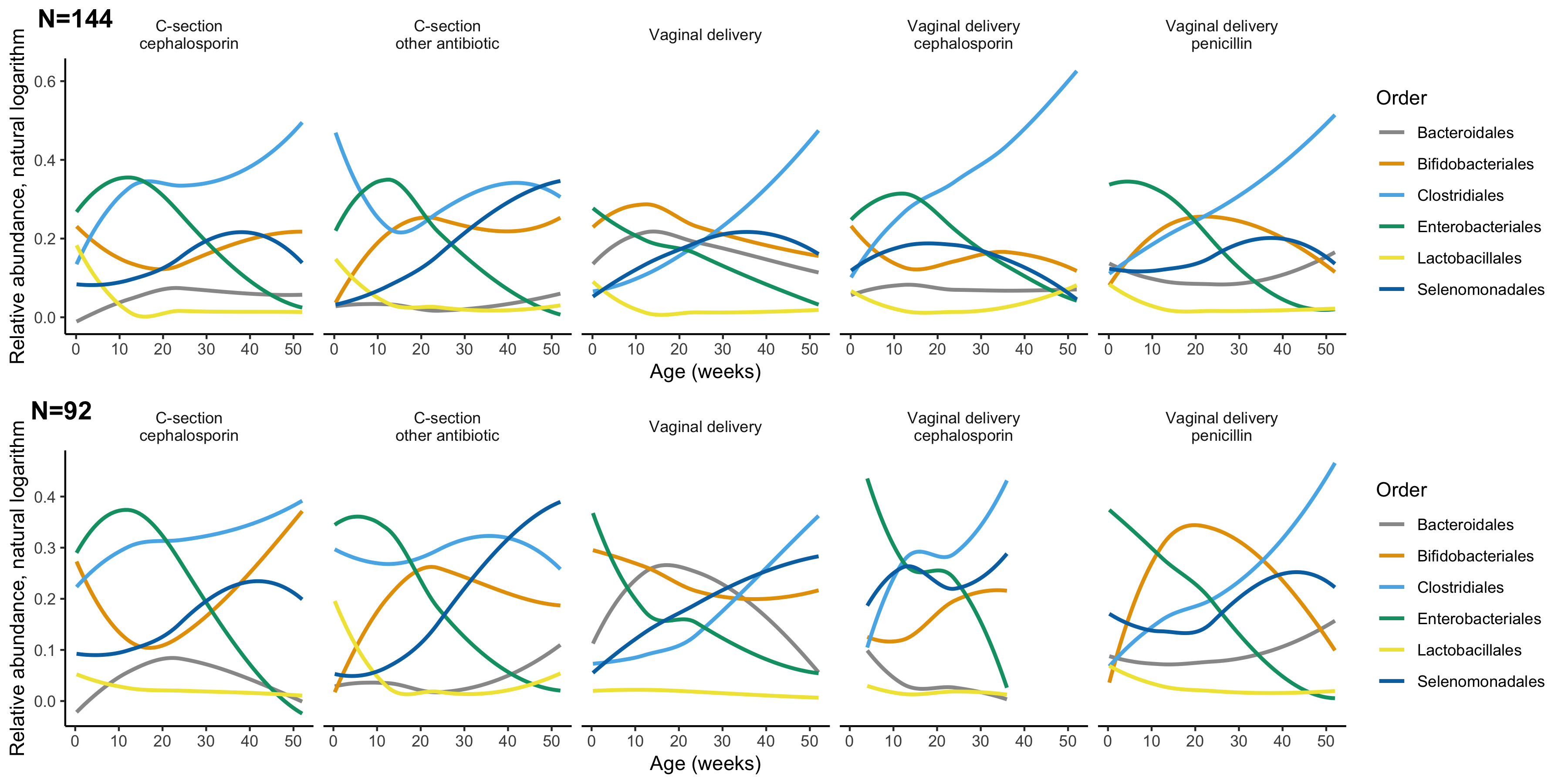

Supplement: Supplemental Material [file KGMI_A_2095775_SM1219.zip › supplementary_figure2.tiff]

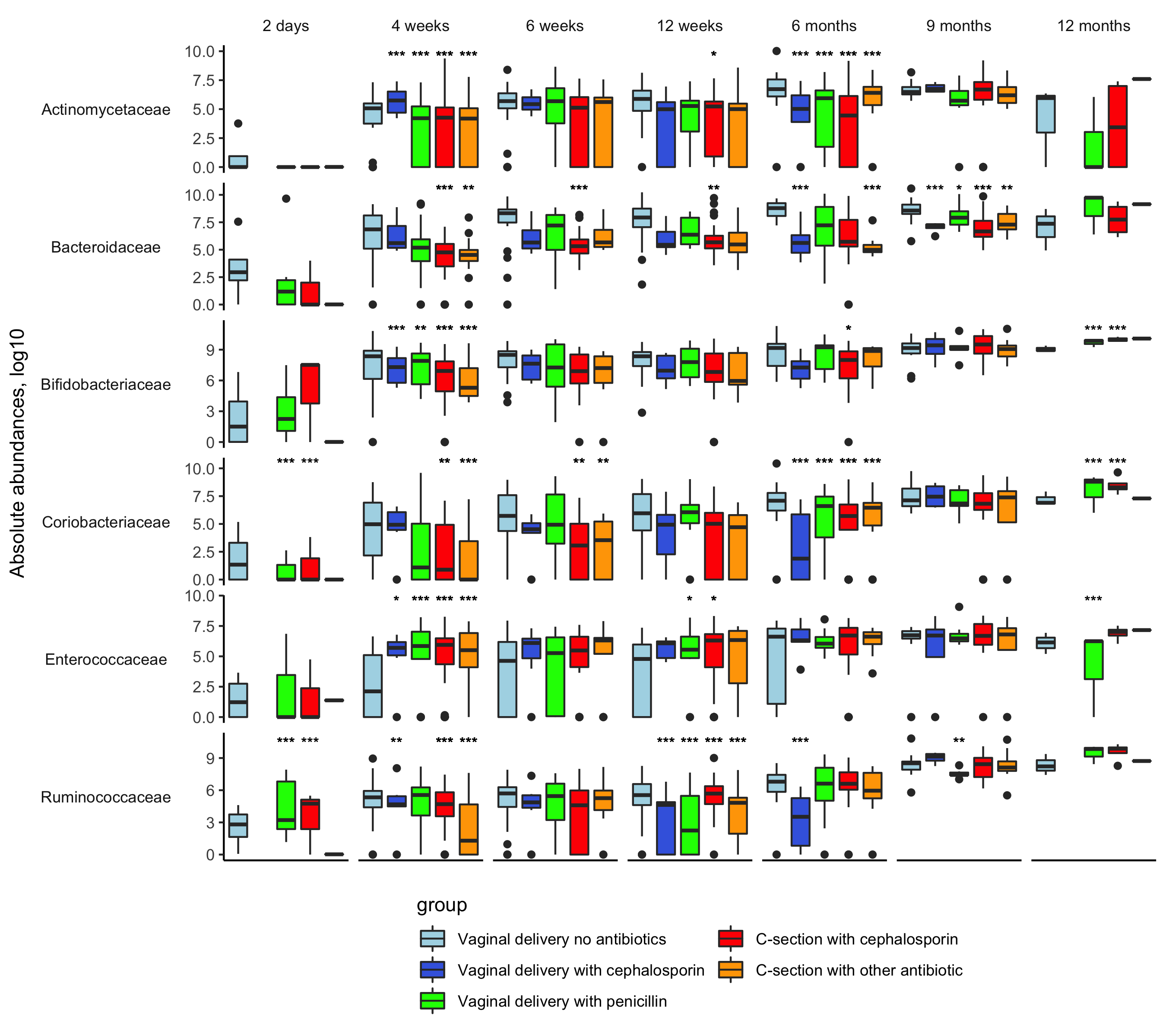

Supplement: Supplemental Material [file KGMI_A_2095775_SM1219.zip › supplementary_figure3.tiff]

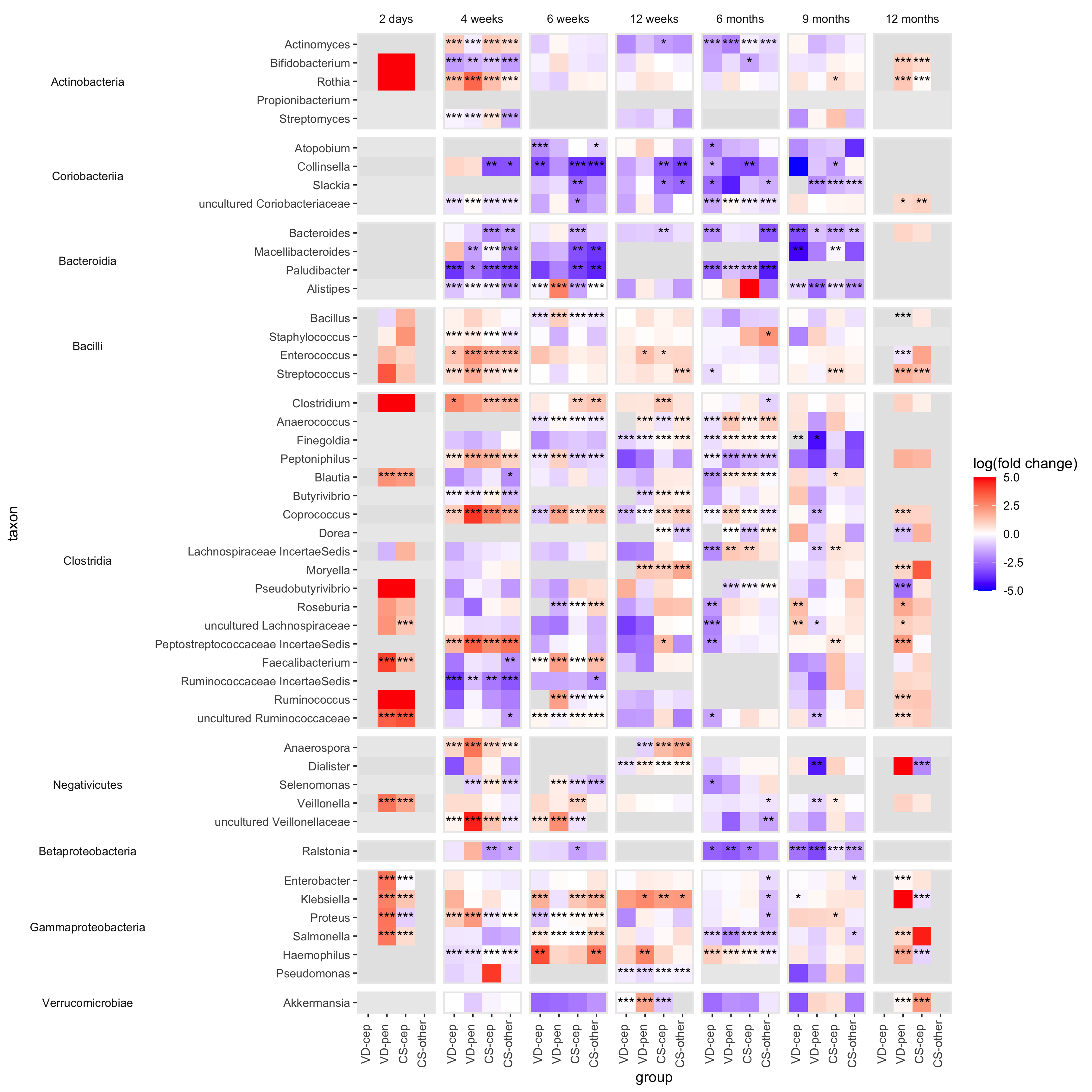

Supplement: Supplemental Material [file KGMI_A_2095775_SM1219.zip › supplementary_figure4.tiff]

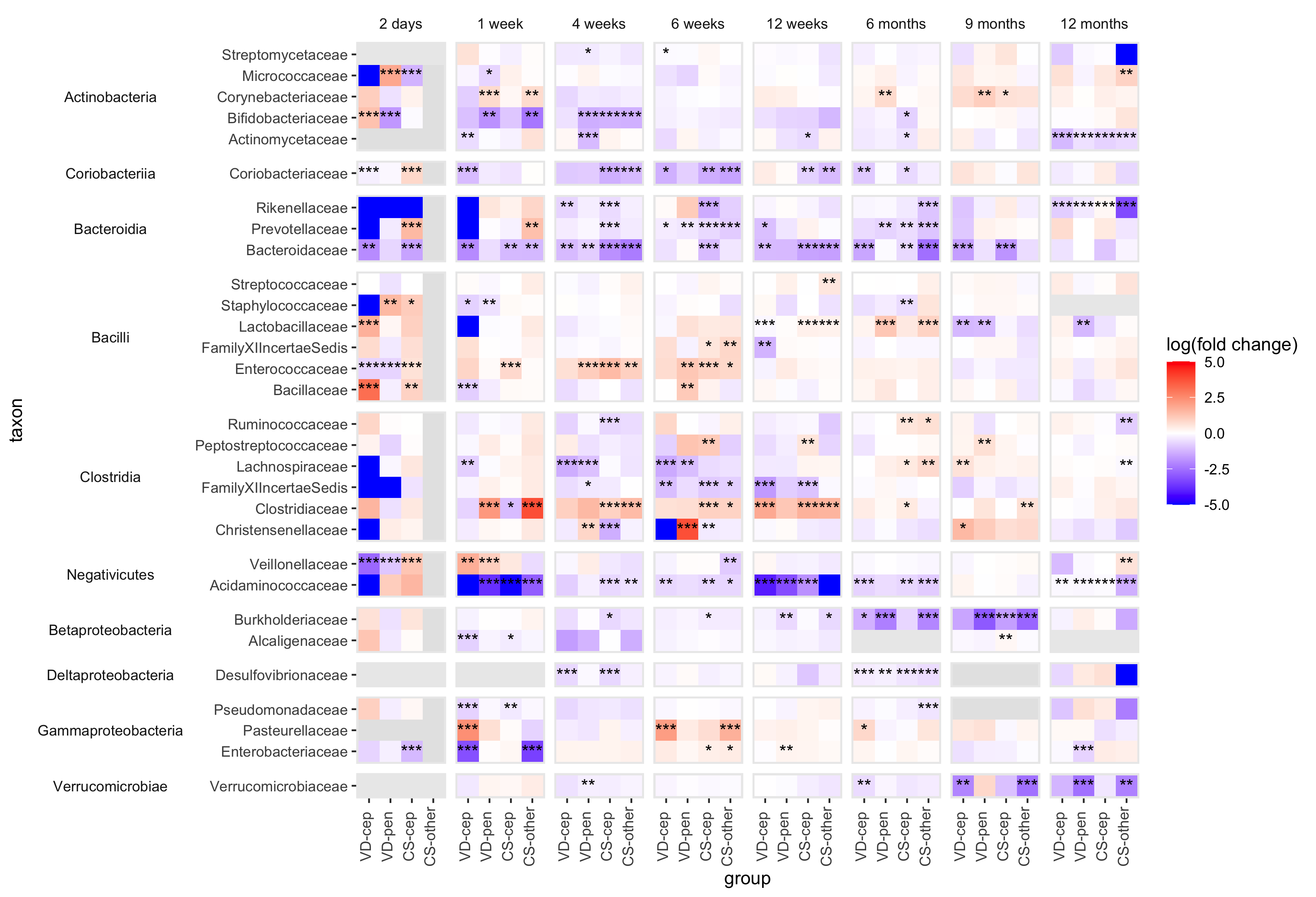

Supplement: Supplemental Material [file KGMI_A_2095775_SM1219.zip › supplementary_figure5.tiff]

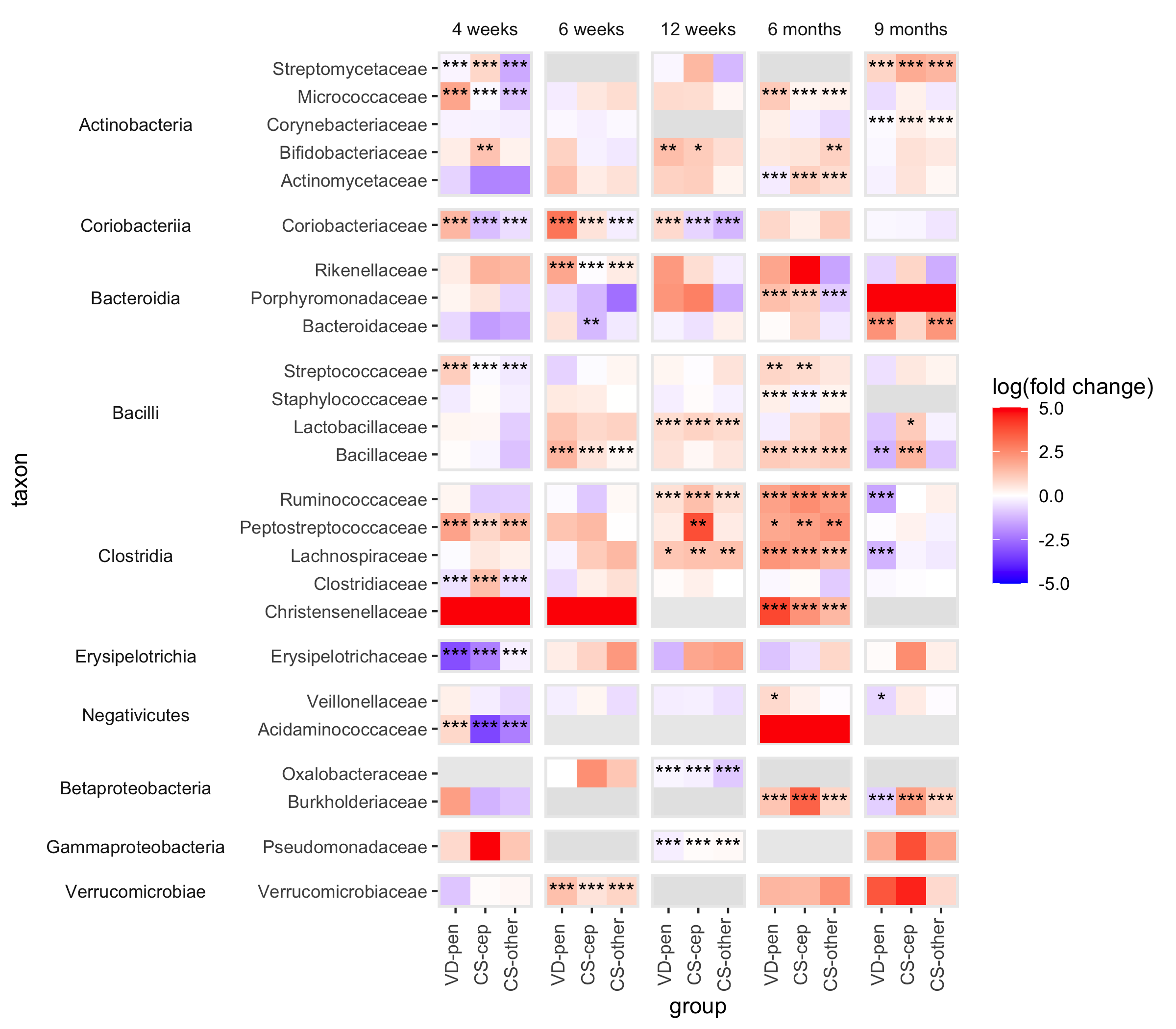

Supplement: Supplemental Material [file KGMI_A_2095775_SM1219.zip › supplementary_figure6.tiff]

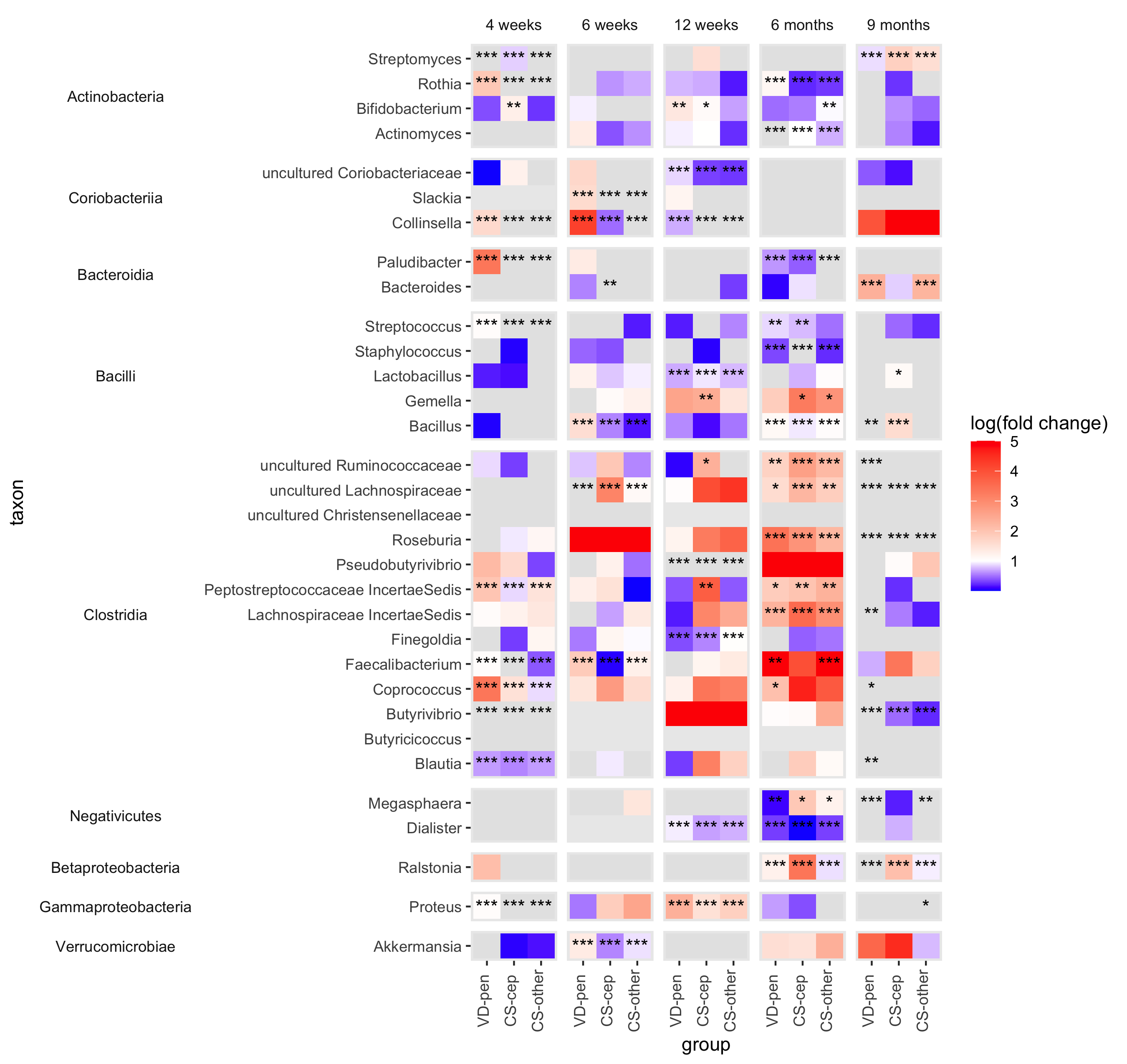

Supplement: Supplemental Material [file KGMI_A_2095775_SM1219.zip › supplementary_figure7.tiff]

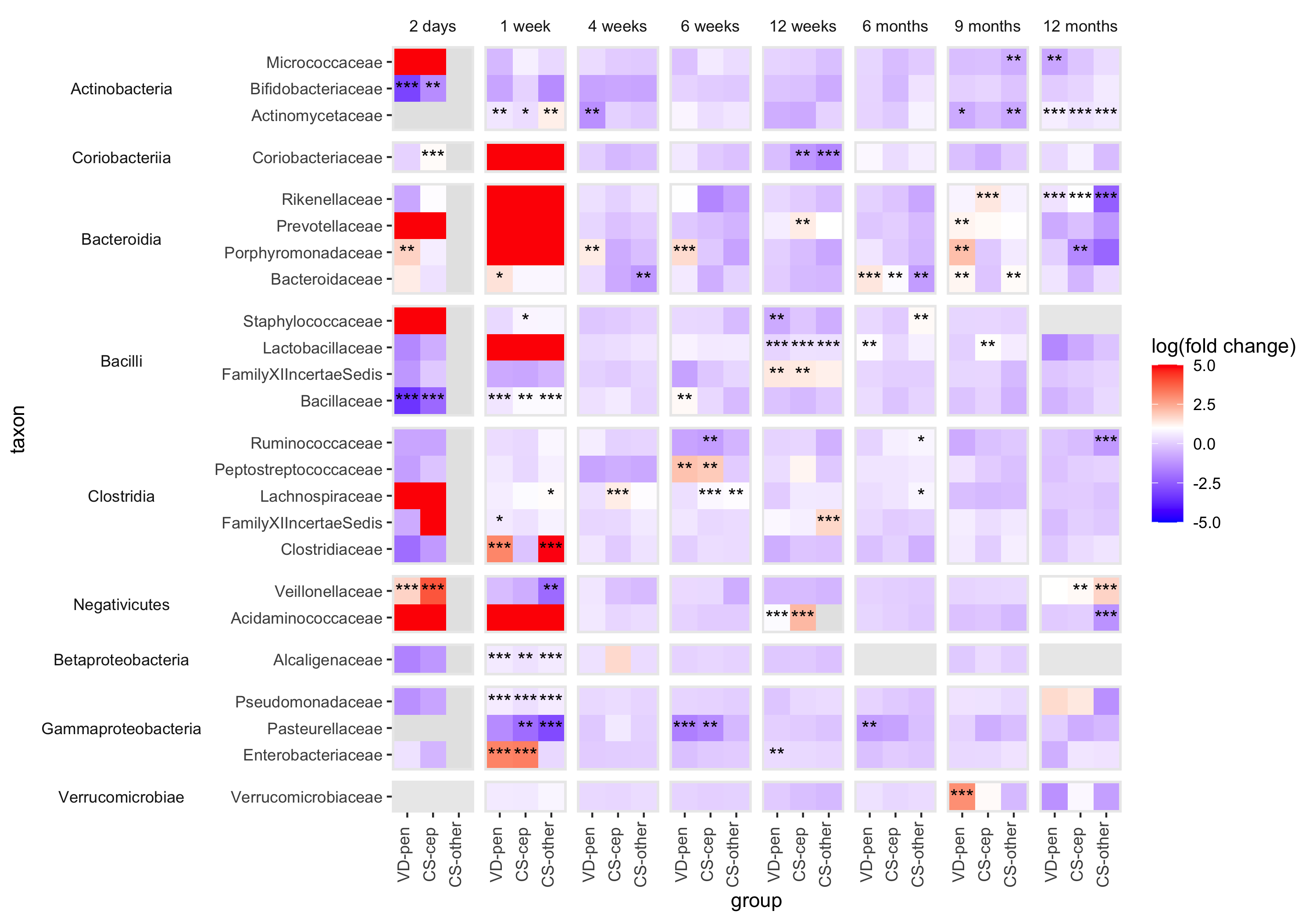

Supplement: Supplemental Material [file KGMI_A_2095775_SM1219.zip › supplementary_figure8.tiff]

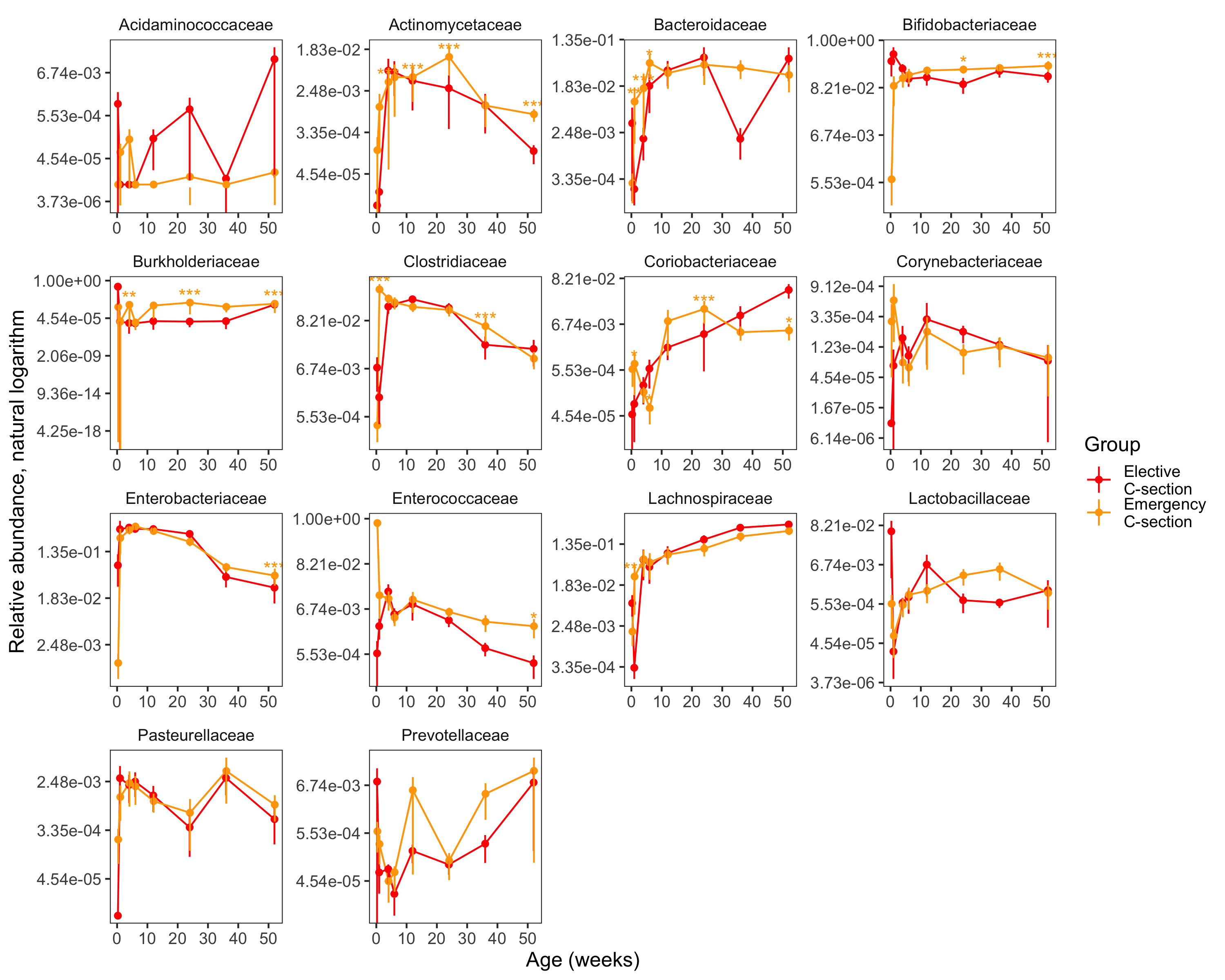

Supplement: Supplemental Material [file KGMI_A_2095775_SM1219.zip › supplementary_figure9.tiff]
